# A standardized lexicon of body odor words crafted from 17 countries

**DOI:** 10.1038/s41597-025-04630-8

**Published:** 2025-02-22

**Authors:** Antonie L. Bierling, Alexander Croy, Fatma Bilem, Leah Bloy, Fiona Yan-Yee HO, Andres F. Jimenez, Pavlína Kyjaková, Mariano Mastinu, Nicole Power Guerra, Uta Sailer, Annett Schirmer, Edgardo C. Silva, Veikko Surakka, Lana Takau, Evelina Thunell, Kedarmal Verma, Barbara R. Żyżelewicz, Asifa Majid, Ilona Croy

**Affiliations:** 1 Department of Clinical Psychology, Institute of Psychology, Friedrich-Schiller-Universität Jena, Jena, Germany; 2 Institute for Materials Science, Technische Universität Dresden, Dresden, Germany; 3 Clinic and Polyclinic for Psychotherapy and Psychosomatics, Faculty of Medicine and University Hospital Carl Gustav Carus, TUD Dresden University of Technology, Dresden, Germany; 4 Institute of Physical Chemistry, Friedrich-Schiller-Universität Jena, Jena, Germany; 5 Department for General Psychology, Institute of Psychology, Friedrich-Schiller-Universität Jena, Jena, Germany; 6 Department of Psychology, Hebrew University of Jerusalem, Jerusalem, Israel; 7 School of Business Administration, Hebrew University of Jerusalem, Jerusalem, Israel; 8 Department of Psychology, The Chinese University of Hong Kong, Hong Kong, Hong Kong; 9 Hotchkiss Brain Institute and Department of Psychiatry, University of Calgary, Calgary, Canada; 10 Schulich School of Medicine and Dentistry, Western University, London, ON Canada; 11 Institute of Organic Chemistry and Biochemistry, Academy of Sciences of the Czech Republic, Prague, Czech Republic; 12 Smell & Taste Clinic, Department of Otorhinolaryngology, Technische Universität Dresden, Dresden, Germany; 13 Department of Behavioral Medicine, Institute of Basic Medical Sciences, Faculty of Medicine, University of Oslo, Oslo, Norway; 14 University of Innsbruck, Innsbruck, Austria; 15 Centre for the Study of Labour and Human Factors, University of Valparaíso, Valparaíso, Chile; 16 Faculty of Information Technology and Communication Sciences, Tampere University, Tampere, Finland; 17 Max Planck Institute for Evolutionary Anthropology, Leipzig, Germany; 18 Department of Clinical Neuroscience, Karolinska Institutet, Stockholm, Sweden; 19 School of Humanities and Social Sciences, Indian Institute of Technology Indore, Indore, India; 20 Institute of Psychology, University of Wroclaw, Wroclaw, Poland; 21 Department of Experimental Psychology, University of Oxford, Oxford, UK; 22 German Center for Mental Health (DZPG), site Halle-Jena-Magdeburg, Halle-Jena-Magdeburg, Germany

**Keywords:** Human behaviour, Human behaviour

## Abstract

Body odors offer a unique window into the physiological and psychological profile of the emitter. This information, broadcast in nonverbal communication, significantly shapes social interactions. However, effectively digitizing body odors requires a precise framework for perceptual operationalization. Previous research has used a very limited number of verbal terms, such as pleasant, intense, or attractive, which fails to adequately capture qualitative differences. To address this gap, we elicited body odor descriptions from 2,607 participants across 17 countries and 13 languages. All these descriptions are presented here in one dataset, together with a condensed list of 25 body odor words (BOW). Those terms reliably differentiated between body states, and were validated in a separate study with a different group of 155 perceivers. The dataset, available as a web application, provides a novel operationalization of body odor impressions, which is a precondition for studying olfaction in human nonverbal communication, for perception-based digitization of body odors and for comparative studies.

## Background & Summary

Social interactions are heavily influenced by the mutual exchange of nonverbal signals, which are broadcast to the perceiver and provide information about many things, including illness, emotional state, and familiarity of the agent. While much research has largely focused on facial signals, the role of body odors for human communication has received much less attention^1^. All physiological and psychological processes in the body, including inflammation, hormonal changes, emotions and genes, are to some degree reflected in a person’s emission of body odors^2^. Hence, body odors inform about health^3^, fertility^4^, emotional status^5^, and kinship^6^.

Although spectroscopic analyses identify changes in human emitted volatiles with increasing precision^7,8^ and allow detection of early olfactory disease markers^9–12^, the richness of olfactory information remains underutilized. This is in strong contrast to visual information where images from facial emotion expressions, for instance, have greatly enhanced our understanding of human communication^13^, and images from disease have led to medical advance^14^. The utility of images is based on the fact that they can be digitally shared across time and space, and the underutilisation of olfactory information is a consequence on the inability to do so.

Digitization requires a transformation of information while at a minimum preserving the percept. Even in a grainy image we can still discern a smile from a frown. However, for olfaction, even the notion of the percept is not precisely defined. Research in body odor perception has predominantly relied on the use of broad evaluative scales such as pleasantness, attractiveness or intensity, e.g., see^15–17^. These scales represent quite an impoverished verbal representation of body odor perception, and the resulting evaluative measures underly high interindividual variability^18–20^. The use of coarse-grained evaluative scales may be a reason why research of body odor perception often shows small effect sizes or inconsistent results^2^. One example is female fertility, with some studies showing that men prefer the body odor of women during ovulation, while others do not find this effect^4,21,22^. The lack of precise perceptual operationalization may also explain why body odors that evoke different behavioral effects are still evaluated as similar^23^.

Probably due to the widely-held (but not universally true) assumption that people lack vocabulary for describing smells^24^, the only notable attempt to apply qualitative descriptors of body odor perception comes from the field of perfumery. An English speaking panel of two perfumers and two fragrance evaluators created 15 basic descriptors (e.g., musty, moldy, earthy, etc.) from smelling 10 out of 62 collected body odor samples and used this list to evaluate the other 52 samples^25^. Six of the fifteen descriptors were commonly used by the expert panel with good intraclass correlation coefficients, and odors from male donors were evaluated different than odors from female donors. While informative, the 15 descriptors cannot simply be applied to lay people’s body odor perception. Experts describe odors differently to lay people (e.g.,^26^), and odor perception varies across populations^27–30^.

The main goal of this study was accordingly to create a robust list of descriptors that enable a precise operationalization of body odor perception. Using a bottom-up approach, we surveyed 2,607 people from 17 countries who described body odors in states of illness, stress, and physical activity, as well as odors from different parts of the body. This approach aimed to provide suitable body odor descriptors in multiple languages. To enable standardized research across languages and to provide a tool for cross-linguistic studies, the most frequent descriptors were collated into a single standardized list, presented here in English. Together, this forms the basis of a final lexicon for body odor words - the BOW. The validity of BOW was evaluated by comparing it to free descriptions given by a separate group of 155 individuals who rated body odors of 61 donors presented to them.

## Methods

### Participants and ethical approvement

Our goal was to collect body odor vocabulary from a variety of countries and languages. To recruit participants, convenience sampling was used on the basis of an existing network of collaborators. The only inclusion criterion was that participants must consider themselves fluent in the language of test in the respective country. Each country or language with at least n = 100 participants who filled in at least one free description was included in the study’s final dataset.

The study was approved by the Ethics Committee of the TU Dresden (process number 361082020) and was conducted according to the principles of the “World Medical Association’s Declaration of Helsinki”. Informed consent from participants to participate in the study and explicit consent for publication of their data in an open access publication was obtained online.

In order to protect the participants’ anonymity, no personal data has been collected or stored together with the dataset. For countries, who chose to give incentives to the participants and needed contact information, the contact data (e-mail addresses) were stored using the functionality of SoSciSurvey, which stores contact data in a separate file unrelated to the raw dataset. After finalizing the study, the contact data have been deleted. In the final dataset, personal data only include age, gender, birthplace and languages spoken from childhood and these are only presented in a separate file, which is not linked to the main dataset (see Data Record 3).

Compensation for participants was in accord with local conventions; no financial compensation was given except for participants from Germany (lottery for five 20–50 EUR vouchers), Norway (voucher with a value of 100 Norwegian Kroner; 9 EUR), Sweden (27 Swedish krona; 2.30 EUR) and Vanuatu (mobile phone cards with a value of 150–200 vatu; 1.0 to 1.5 EUR). In Germany, Austria and Finland, psychology students could receive subject compensation hours.

### Materials and Procedure

The online study consisted of four main parts and had a total duration of approximately 15 minutes. First, before completing the main part of the study, participants were asked to indicate their age, gender, country of birth, and languages spoken since childhood. Second, participants were asked to: “Please write at least three words” describing body odors for healthy, sick, and exercising individuals; as well as from different parts of the body, i.e., the mouth, armpit, feet, female genital and male genital area. Participants could provide a maximum of five descriptions. If participants were unable to find three descriptions, they were prompted that they had not filled in all fields, but they were permitted to continue the survey if they ticked a box that indicated they did not wish to provide further descriptions. Please note that descriptions were given without smelling actual body odor samples. The rationale for this decision is given in the section Validation study. Third, participants indicated, from which body parts they perceive body odor. This was done by showing participants a sketch of a gender neutral silhouette of a person, once from the front and once from the back (silhouette images as in^31^). Participants were then instructed to mark those body parts, from which they perceive body odor by clicking on the respective body parts. There was no limit to how many body parts could be marked. This task was conducted twice, once asking for the participants’ own body odor and once asking for the body odor of other persons. And finally, in the fourth part, participants were asked to report on their smell behavior in their everyday lives using a questionnaire developed by Perl, Mishor *et al*. (2020). The full English questionnaire is provided (see Data Record 4). Since the focus of this manuscript is the dataset of the body odor lexicon, the data from the third and fourth tasks are not considered further. The English survey was distributed to each collaborator and if necessary, translated into the local language. The original English and translated versions were implemented in SoSciSurvey, an online survey platform. A link unique to each collaborator’s recruitment site was generated and distributed to prospective participants.

### Data Preprocessing

Several preprocessing steps were conducted with the raw data. For each language, all free descriptions, for all questions (all four states - healthy, sick, stressed, after exercise; and body odor sources - armpit, mouth, feet, male and female genitals) were collated into one long list of descriptions, sorted alphabetically, and the frequency of each unique entry was calculated (e.g., all mentions of *sweaty*). The responsible researcher for data collection in each language site then received their language’s dataset and went through the list manually to identify the citation form of the term, i.e., the dictionary form of each word (also known as, “lemmatization”). First, different spellings or alternates of the same word were identified and standardized (e.g., *sweaty*, *sweatie*, *sweat* -> *sweaty*). Next, complex entries like *this smells sweaty and kind of sweet* were coded into their component parts, e.g., *sweaty* and *sweet*. Detailed instructions are provided in the original lemmatization instructions, see Data Record 4. Once the coding was finalized, the frequencies for each term were re-calculated and the lists for each language sorted by frequency. These lists were then used to identify the most frequent terms to describe body odors across languages, as outlined in the next section.

### Creation of a cross-linguistic list of body odor terms

Across all 13 languages, a total of 60,275 raw descriptions were given by the 2,607 participants, from which we identified 7,489 unique descriptors. Only a few descriptors were elicited across many participants, instead a great number of terms were listed infrequently and by only a handful of participants, resulting in the common Zipfian distribution for this sort of data. To focus on common vocabulary, for each language we included the most frequently elicited descriptors for further processing, aiming to cover at minimum 50% of participants’ original descriptions per language. For example, 989 unique standardized descriptors were identified for the German dataset from the original 13,360 tokens. However, the 17 most frequent word types already covered roughly 6,600 of the original description tokens, corresponding to 51.1% of all German responses. The remaining 972 unique descriptor types thus represent the “tail” of the Zipfian distribution, consisting of many infrequent terms. The language with the highest cumulative word count was Chinese, with 42 words representing the 50% most frequent terms in that language. Accordingly, we set the cut-off at that number for all languages and identified the 42 most frequent words from each language. For some languages, several terms had identical number of mentions at or around rank 42 (e.g., in Polish, four terms shared rank 42 with 13 mentions each). In that case, all words were considered. The words were then translated into English using a back-translation procedure. Any discrepancies from the back-translation were resolved by discussion. The English words were then again standardized, if necessary, as described in the previous section (e.g., the Norwegian word *fisk* is translated as *fish*, and further standardized as *fishy*). This resulted in a final list of 192 English descriptors, which covered 39,846 (66.1%) of the original responses. In the next step, we reduced this list of 192 descriptors and identified common vocabulary across languages while still preserving the representation of each country. So, all words that fulfilled the following criteria were included: (a) the term was top five in at least one country AND in the top 42 position in five or more other countries OR (b) the term was top ten in at least one country AND in the top 42 position in ten or more other countries. This resulted in a final list of 25 terms (see Fig. 3).

### Validation: Independent body odor perception study

Since the study was online, participants gave body odor descriptions without smelling actual odorant samples. The reason is, that body odor samples, which are typically collected as axillary sweat on cotton pads^32^, are very sensitive and must therefore be stored and transported frozen. They must be thawed before the odor presentation, but lose in intensity with each thawing process, which is why samples are typically only thawed four times^32^. Hence, it was not feasible logistically to provide the same body odor samples to such a large sample of participants distributed over 17 countries, including Vanuatu, which is more than 15,000 km away from Germany. In order to check the comparability of generating words in a language-only task as implemented here to generating words after smelling body odors, we validated our approach. Therefore, we used axillary sweat samples of healthy individuals, which have originally been collected for two other body odor perception studies conducted by AB. The first study, “body odor states”, focused on investigating perceptual differences in body odors collected from 40 healthy young males (aged 18–40 years, *M* = 23.8, *SD* = 4.7) that were each in a control, stressed, sexually aroused, and exercise state (Bierling *et al*., *in preparation*). For our validation purpose, only body odors from the control condition have been used. The samples from always ten of the forty donors were pooled and combined to four donor pools. The second study, “body odor traits” focused on cross-sectional differences of perception between n = 21 healthy female individuals (aged 19–61 years, *M* = 32.1, *SD* = 11.5; Bierling *et al*., *in preparation*). In this study, body odor samples were presented individually, not pooled. Thus, the descriptions used for validation were obtained for a total of 25 body odor samples (four pooled plus 21 individual), obtained from 61 healthy individuals.

As both studies have very similar procedures of donation and perception, they are presented jointly. The 61 body odor donors sat for 40–50 min in a heated room while wearing cotton pads (Hans Natur, 3-layer 100% organic cotton nursing pads, obtained from Hans Natur online store) in the axillar region. The donation followed a strict hygiene protocol, including instructing participants to avoid perfumed hygiene products and strongly spiced foods, onions, garlic, leeks, asparagus, cabbage and alcohol, and refrain from smoking for at least 12 hours before testing. At the appointment, the participants were asked to wash their axillary regions in our laboratory using an odourless medical shower gel. After washing, the cotton pads were attached to the participants’ axillar regions, and they were asked to put on a 100% cotton t-shirt provided by us. The cotton pads and the t-shirts were pre-washed using an odorless detergent. While wearing the cotton pads, the participants filled in a series of questionnaires, screening for medical status and mental health. For validation, we only included the 61 donors (*n* = 18 donors excluded) who indicated no significant health impairment and who reported no above-threshold depressive, anxiety or somatic symptoms (values < 9) in the Patient Health Questionnaire (PHQ-D)^33^. Participants furthermore completed questionnaires on socio-demographic data, odor habits, personality, and affective state, which are outside the scope of the validation. After donation, the cotton pads were cut into four equal sizes (8 snippets per person) and stored in labelled resealable plastic bags at −25^°^C in a laboratory freezer. For “body odor states”, snippets were pooled, i.e. each presented body odor sample contained 10 snippets from 10 different male donors (4 samples in total). For “body odor traits” each presented body odor contained three snippets from the same female donor (21 samples in total).

The 25 samples were rated by *n* = 155 independent perceivers (“body odor states”: *n* = 78, aged 18–28 years, *M* = 21.3, *SD* = 2.4 91.1% female; “body odor traits”: *n* = 77, aged 18–60 years, *M* = 25.7, *SD* = 8.3, 76.7% female). Each perceiver was asked to give a free description per sample using the same general instructions as in the online study (“Please write at least three words or phrases describing this sample”). From the 155 perceivers, we received a total of 5,338 free descriptions, which were again clustered, standardized and translated into English using the same procedure as described in Section “Data Preprocessing”. This resulted in 605 unique terms. We then calculated the percentage of terms which overlap with the 25 descriptors identified from the cross-linguistic study. For that purpose, we searched for all 25 cross-linguistically identified terms within the 605 descriptors of the validation study, and calculated how often those were produced by the body odor perceivers given the total number of responses.

### Analyses and visualization

For the technical validation, we investigated differences in the usage of BOW terms between body states and body odor sources. For that purpose, we conducted *χ*^2^ homogeneity tests. Significance level was set at p < 0.05, with correction of p-values for multiple testing applied. Effect size was estimated using Cramer’s V. All analyses and visualizations were carried out in Jupyter Notebook using Python 3.7 and the packages pandas, NumPy, pingouin, SciPy, seaborn and matplotlib. Further edits of images and illustrations were conducted using Adobe Illustrator 2025.

## Data Records

### Datasets

The full dataset, as well as all material used for this study is available at the repository of the Open Science Framework (RPZJK)^34^.

#### Data Record 1 - dataset main study

The main dataset is **bow**_**dataset.xlsx**^34^. The file contains the processed dataset including the original free descriptions together with the lemmas and translations of all top words (see methods section for selection of top words). The column “id” contains a consecutive number identifying each individual study participant. Furthermore, the “age” in years and the “gender” (male, female, non-binary, other) of the participants are provided. The columns “country” and “language” refer to the country and language of recruitment. The column “condition” refers to the four body states (healthy, sick, stressed, after exercise) and five body odor sources (from the armpit, from the mouth, from the feet, from the female genitals, from the male genitals). The column “full description” contains the unprocessed original description, i.e., without preprocessing, corrections or similar. The column “lemma” provides the mapped dictionary form, i.e., the standardized terms given for the full description. Note that there can be several lemmas for one full description. The dataset is given in the long format, thus, for each study participant and each full description there are as many rows as unique lemmas exist. The column “translation” provides the back- and forth translation results for all 42 top words of each language. These were again standardized for better comparability between languages (as described in section 2.4). The resulting standardized translations are given in “translation_lemmatized”.

Based on the described dataset, we have built an interactive web application available at https://bow-descriptors.streamlit.app/. Via the app, the most frequently named terms can be filtered by language, country (for those languages recruited in several countries), body state, and body odor sources.

#### Data Record 2 - dataset validation study

The dataset of the validation study is called **dataset**_**validation.xlsx**^34^. One row in the dataset represents one description (column “description”) by one perceiver participant (solumn “code_perc”) for one sample (column “sample_name”). There can be several rows for the same perceiver and sample, as participants were allowed to give several descriptions. The file contains five columns: The first column “study” refers to which of the two body odor studies these descriptions have been derived from (“states” or “traits” study; details on the two studies are discussed above in the method section on the validation study). The second column “code_perc” is an identifier for the perceiver participant giving the free descriptions. The third column “sample_name” refers to the different samples, i.e., pools in study “states” and individuals in study “traits”. The fourth column “description” contains the standardized free descriptions given by the 155 German perceivers. Note that the German descriptions are provided all in lower case as a result of the preprocessing steps on the descriptions (see methods). The fifth column “translation_lemmatized” gives the standardized English translations for each description in analogy to the standardized translations in the main dataset.

#### Data Record 3 - sociodemographic variables

In order to protect the anonymity of our participants, the sociodemographic variables are stored in another dataset called **dataset**_**sociodemographics.xlsx**^34^. The first column “id” is an identifier distinguishing the study participants. Please note that the id does **not** match with the “id” variable from the main dataset due to our data protection policy. The columns “country” and “language” refer to the country and language of recruitment. The column “age” refers to the participant’s age in years, “gender” to the gender given in a drop-down format (male, female, non-binary, other). The column “birthplace” refers to the country of birth. Some participants gave their birth date instead. These entries have been omitted. The column “spoken_languages” gives the languages spoken by the participants from childhood.

#### Data Record 4 - further files

As further material, we provide the English questionnaire as PDF, the lemmatization instruction and an English and empty template version of the multiple choice matrix displayed in Fig. 3. All files are available in the folder “Info and Material”^34^.

### Sample characterization

**Fig. 1:**
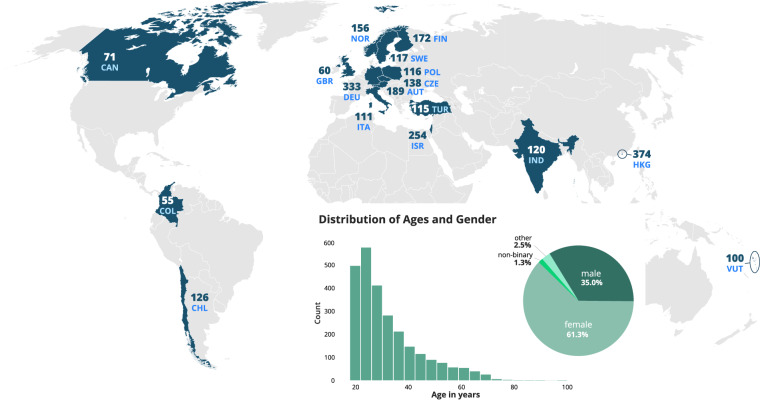
Demographics of the 2,607 study participants who provided data for the body odor word list (BOW), including country of test, age, and gender.

**Fig. 2:**
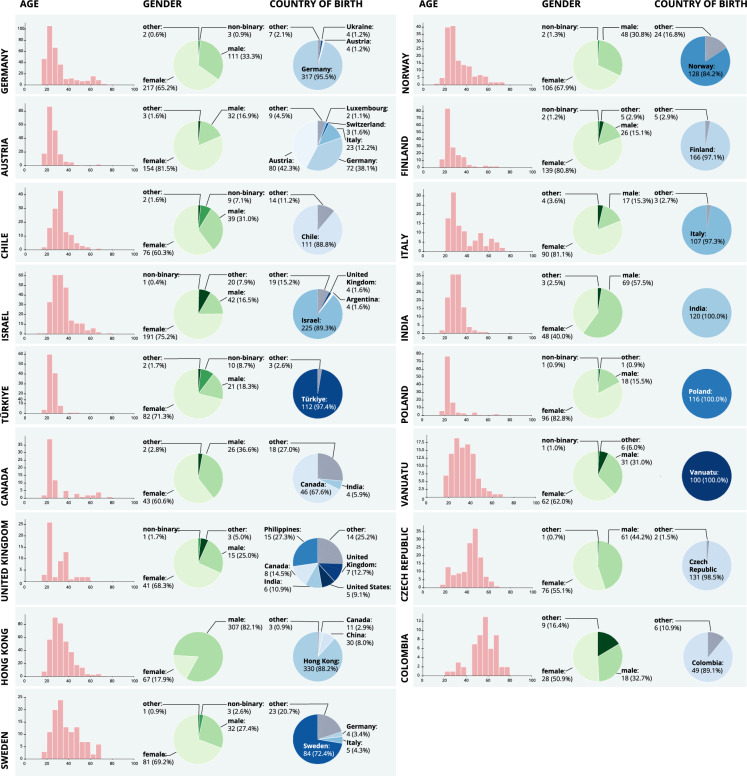
For each country, the distribution of age (y axis displays absolute count) in years, as well as the percentages of gender and birthplaces are given. Note that for birthplace for the sake of readbility only categories are displayed, which represents more than 3 individuals and more than 1% of the population. The full dataset of sociodemographic variables is available, see Data Record 3.

The final sample contains body odor vocabulary from 17 collection sites, who recruited participants speaking 13 different languages (German, English, Spanish, Italian, Polish, Czech, Norwegian, Finnish, Turkish, Hebrew, Chinese, Bislama, Hindi; see Fig. 1). For three languages, several countries recruited participants: Austria and Germany recruited in German; the United Kingdom, Canada, and Sweden in English; and Chile and Colombia in Spanish. From the total 2,607 participants, there were 1597 females (61.3%), 913 males (35.0%), 33 non-binary participants (1.3%), and 64 participants who identified as “other” (2.5%). Their age ranged from 18 to 83 years, with a mean age of 32.24 years (SD = 11.92); see Figs. 1, 2 and Table 1 for a breakdown of demographics and recruitment by country.

**Table 1:** Overview of recruited participants per country and language.

country	N per country	language	N per language
Germany	333	German	522
Austria	189		
Hong Kong	374	Chinese (trad.)	374
Israel	254	Hebrew	254
United Kingdom	60	English	248
Canada	71		
Sweden	117		
Chile	126	Spanish	181
Colombia	55		
Finland	172	Finnish	172
Norway	156	Norwegian	156
Czech Republic	138	Czech	138
India	120	Hindi	120
Turkey	115	Turkish	115
Italy	111	Italian	111
Poland	116	Polish	116
Vanuatu	100	Bislama	100

**Fig. 3:**
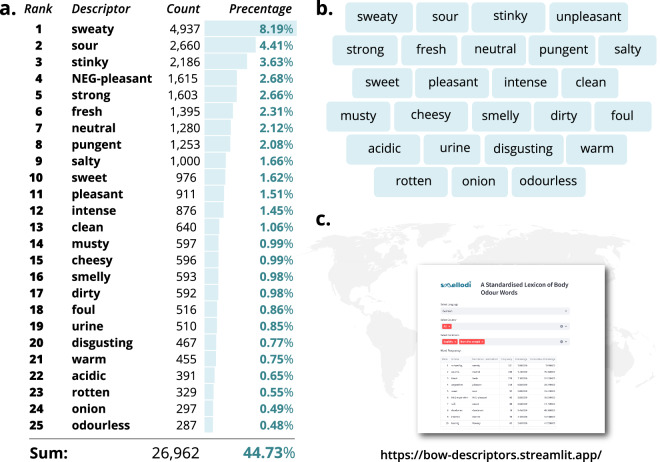
**a**. The 25 most frequent words used to describe body odors across languages ranked by frequency. Counts and percentages relate to the total number of 60,275 descriptions given across all speakers. “NEG” is used for any negation, e.g., “NEG-pleasant” includes both unpleasant and not pleasant. **b**. The top 25 BOW descriptors arranged in a two-dimensional multiple-choice matrix. This arrangement is suggested as a means of efficiently presenting BOW to participants in future perception tasks. The matrix is available in English as well as in an empty form (see Data Record 4). **c**. Language specific lists can be obtained from a web application accessible via https://bow-descriptors.streamlit.app. Shown here is a screenshot from the application. The web application enables users to filter the full dataset by language and country (for languages recruited in several countries), as well as by body states and body odor sources. The most frequent terms for languages and conditions can be downloaded as a .csv file.

## Technical Validation

### Application and representativity of the dataset

**Fig. 4:**
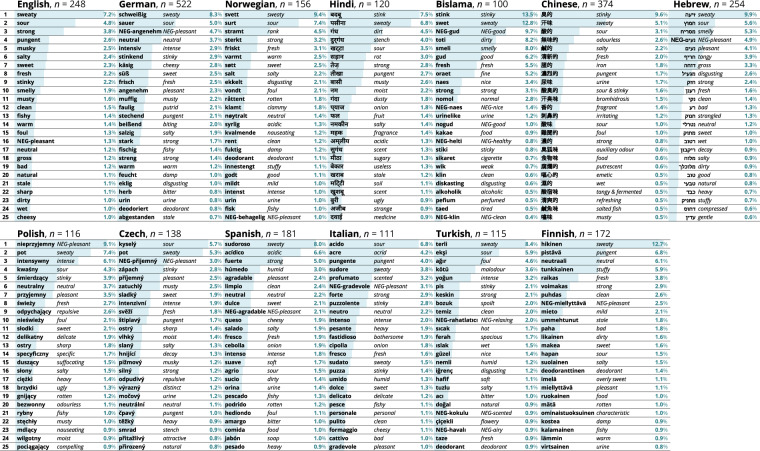
Twenty-five most frequent terms in each of the 13 languages, with the English translations and frequency (relative occurrences across states and body odor sources). Participant numbers are provided next to country names.

**Fig. 5:**
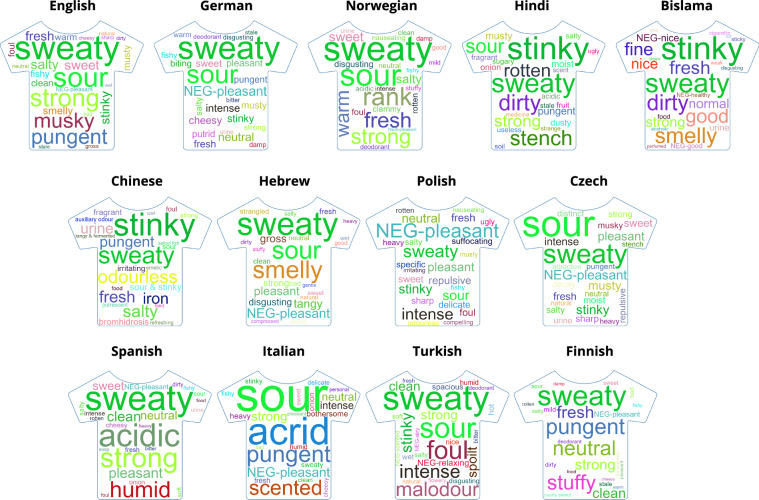
For better comparison between the most frequent terms across the different languages, here we display the twenty-five most frequent terms in each of the 13 languages, translated into English, as word clouds. Each word cloud illustrates the frequency of each of the 25 most frequent terms in the respective language by the size of the term. The same terms across languages are highlighted in the same color. For comparison of absolute frequencies and percentages please refer to Fig. 4.

Across all languages and conditions, we derived a total number of 60,275 body odor descriptors with 7,489 unique terms. We provide these descriptors in language specific lists (see Figs. 4 and 5) and one standardized list of 25 words presented in English (see Fig. 3), which represents 44.7% of the total descriptors. Together they form the lexicon of body odor words - BOW. Thus, BOW provides researchers with a toolbox of qualitative labels which can be applied in studies using the respective language’s list in order to address cultural differences in odor perception^28,35^. In addition, the curated standardized list of 25 English descriptors contains the most common terms across countries while maintaining overall representativity and can be used where cross-cultural comparisons are envisioned, or for countries whose language is not represented in BOW. In order to extend the range of BOW, we encourage groups interested in generating a body odor lexicon for additional languages to get in touch with us.

### Body odor word usage for different states and body odor sources

**Fig. 6:**
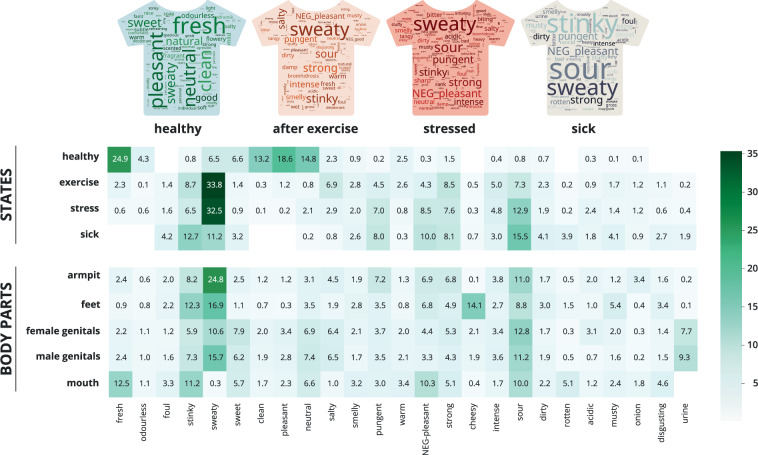
Heatmap of the 25 BOW descriptors (bottom row) per condition for both state (top panel) and body odor sources (bottom panel) as a percentage of the total mentions. Word clouds illustrate the distribution of descriptors for each state (based on 194 translated words). “NEG” means negation, e.g., NEG pleasant = unpleasant or not pleasant.

To check if BOW can distinguish between the investigated states and body odor sources, we compared the pattern of using the 25 English standardized descriptors. Healthy body odors overall showed a different response pattern to the other three conditions (see Fig. 6). The most frequent descriptor for healthy body odors was *fresh* with 821 mentions, which covers about a quarter (24.9%) of total mentions from the 25 BOW descriptors, followed by *pleasant* (18.6%), *neutral* (14.8%), and *clean* (13.2%). For exercise and stress odors, the dominant descriptor was *sweaty* with more than 1,000 mentions covering roughly one third (33.8% and 32.5%) of all mentions, followed by *stinky* (8.7%), *strong* (8.5%) and *sour* (7.3%) for exercise, and *sour* (12.9%), *unpleasant* (8.5%), *strong* (7.6%) and *pungent* (7.0%) for stress. Interestingly, sick body odors showed the most distributed pattern, i.e., many descriptors showed similar frequencies, but also several descriptors were hardly used at all. The highest frequency descriptor was *sour* (15.5%), followed by *stinky* (12.7%), *sweaty* (11.2%), unpleasant (10.0%), strong (8.1%) and pungent (8.0%). However, there were limited uses of *fresh* (1), *odorless* (3), *clean* (0) or *pleasant* (1) - all less than 0.1%. Accordingly, *χ*^2^ tests revealed significant differences in the use of descriptors between the four conditions, with a large effect size (Cramer’s *V* = 0.51). Pairwise comparisons showed the biggest difference between healthy and sick conditions, with a moderate effect size (Cramer’s *V* = 0.35), followed closely by healthy vs. exercise, and healthy vs. stress (Cramer’s *V* = 0.33 and 0.32). The comparisons between sick, exercise, and stress all showed significant differences, but with small effect sizes (Cramer’s *V* = 0.09 to 0.18).

Regarding body odor sources, *sweaty* was the most frequent descriptor for the armpit (24.8%), feet (16.9%), and male genital area (15.7%). Body odor from the mouth was most often described as *fresh* (12.5%) or *stinky* (11.2%), body odor from the female genitals as *sour* (12.8%), followed by *sweaty* (10.6%). Overall, *sour* was used as second or third most frequent word for all body odor sources, except for *feet*. *Urine* was almost exclusively used for female/male genitals (7.7% and 9.3%), and *cheesy* was almost exclusively used for feet (see Fig. 6). In line with this, *χ*^2^ homogeneity tests revealed significant differences in the use of descriptors between body odor parts, with a moderate effect size (Cramer’s *V* = 0.29). All pairwise comparisons showed significant differences with moderate to large effect sizes. The largest effect size was for the comparison between mouth vs. feet (Cramer’s *V* = 0.50), and the smallest difference was between male and female genitals (Cramer’s *V* = 0.14).

### Validation study

**Fig. 7:**
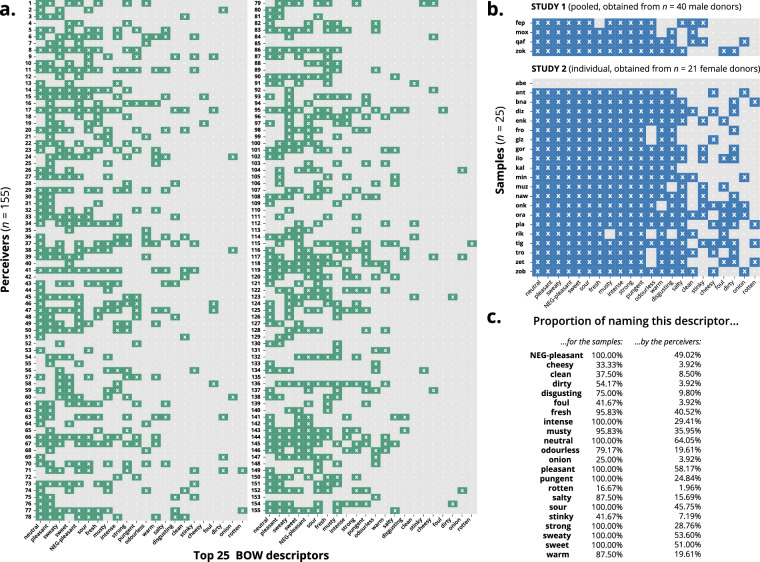
Heatmap showing the usage of the 25 BOW descriptors (x-axis) per perceiver (**a**.) and per sample (**b**.) in the validation study. An “x” marks that the term has been used at least once by this perceiver, “-” indicates no usage of this term for any smelled sample. The percentages of samples the descriptors are used for, as well as the percentages of perceivers which used the terms are given in panel **c**.

As described above, for validation purposes, we compared BOW to the free descriptions obtained from a group of 155 separate perceivers, who smelled at 25 body odor samples obtained from 61 healthy body odor donors. As a result, out of the 5,338 free descriptions, 2,284 descriptions were represented by 22 of the 25 BOW words, hence there was an overlap of 42.8%. Figure 7 shows the frequency of using each of the descriptors (at least one time) by each perceiver and for each sample. Some descriptors were very typically used by many perceivers and for almost all samples, such as *neutral*, *pleasant* and *sweaty*. Other terms were less frequently used, e.g., *onion* and *rotten* (see Fig. 7). The two BOW descriptors, which the participants did not use were *urine*, *smelly* and *acidic*. In addition to the words covered by BOW, the validation study revealed a few other dominant words (see Data Record 2), such as *light* which ranked position 7 with 159 mentions (3.0%), *subtle* ranked 9 (131 times, 2.5%) and *old* ranked 12 (109 times, 2.0%). These words were also present in BOW, but at lower ranks.

## Usage Notes

We identified three possible ways of using data from BOW.

1) For researchers, who wish to use the most frequent BOW descriptors to implement them in their body odor research, we recommend to use either their language’s list from the 13 language-specific lists provided in Fig. 4 or the curated standardized list (Fig. 3) if cross-cultural comparisons are envisioned or if the respective language is not available in BOW. If researchers want to include only descriptors for specific states or body odor sources, all language lists with frequencies can be sorted and filtered via our interactive web application https://bow-descriptors.streamlit.com/. A suggestion on how to present a multiple choice matrix of 25 terms in body odor perception studies is provided as a .png file, see folder “Info and Material” at 10.17605/OSF.IO/RPZJK.

2) Researchers who want to conduct further analyses with the BOW dataset can download the full processed dataset “bow_dataset.xlsx”, which is described in Data Record 1 and is available at^34^. In addition to the processed dataset, we also provide all raw datasets and code. The jupyter notebook “bow_analyses.ipynb” can be used to rerun all analysis steps described in this work. Detailed instructions are given in the notebook. In a second jupyter notebook “bow_preprocessing.ipynb”, the preprocessing of the raw descriptions can be replicated. In order for all code to work, please download the full folder “Analysis with Jupyter Notebook” with all files from^34^.

3) Researchers who want to add their language to BOW are advised to get in touch with the corresponding author of this paper. We provide researchers with the original questionnaire (in English) for translation and distribution in the new language. In order to add further languages to BOW, the file of collected descriptions must be provided in the same format as the full dataset. An example file ("dataset_example.xlsx”) is given in the folder “example files”. New data must then be preprocessed using the analysis script in “bow_preprocessing.ipynb” and manually standardized using the lemmatization instruction provided in “Info and Material”. Further instructions are given in the jupyter notebook and the lemmatization instruction.

## Data Availability

All code and files are accessible via^34^. The code for the web application is accessible via https://github.com/albierling/bow-descriptors.
